# Anthropic pressure and causes of death of stranded *Chelonia mydas* along the northern coast of Bahia - Brazil

**DOI:** 10.1371/journal.pone.0338512

**Published:** 2026-01-27

**Authors:** Danielle Nascimento Silva, José Luís Catão-Dias, Wendell Marcelo de Souza Perinotto, Thaís Pires, Gustavo Rodamilans Macedo, Priscilla Carla dos Santos Costa, Lorena Ferreira Oliveira, Paula Velozo Leal, Alessandra Estrela-Lima

**Affiliations:** 1 Postgraduate Program in Animal Science in the Tropics, Federal University of Bahia, Salvador, Bahia, Brazil; 2 Laboratory of Wildlife Comparative Pathology, School of Veterinary Medicine and Animal Sciences, University of São Paulo, São Paulo, São Paulo, Brazil; 3 Center of Agrarian, Environmental and Biological Sciences, Federal University of Recôncavo da Bahia, Cruz das Almas, Bahia, Brazil; 4 Fundação Projeto Tamar, Mata de São João, Bahia, Brazil; 5 Projeto Baleia Jubarte, Mata de São João, Bahia, Brazil; 6 Instituto de Pesquisa e Reabilitação de Animais Marinhos – IPRAM, Vila Velha, Espírito Santo, Brazil; 7 Department of Veterinary Anatomy, Pathology and Clinics, Federal University of Bahia, Salvador, Bahia, Brazil; University of Mississippi, Oxford, United States of America

## Abstract

This study describes the causes of mortality and the primary pathological findings in 61 sea turtles of the species *Chelonia mydas* that were stranded on the northern coast of Bahia, Brazil. Macroscopic and microscopic lesions were compiled to describe the pathological findings in turtles that were found dead on the beach or that died during treatment at the rehabilitation center of the Fundação Projeto Tamar. Among the 61 turtles evaluated, the cause of death was determined in 96.7% (59/61) of cases. These turtles were affected by natural threats (of infectious, neoplastic, and metabolic origin) and anthropogenic interactions, the latter represented by fishing and/or the presence of anthropogenic waste. 3.3% (2/61) of the cases were classified as inconclusive as to the cause of death, as they were euthanized. Acute respiratory failure was the main cause of death, affecting 45.8% (27/59) of the animals, followed by sepsis in 28.8% (17/59), cachexia/malnutrition in 16.9% (10/59), circulatory collapse due to thromboembolism in 6.9% (4/59), and one animal (1.6%, n = 59) had death associated with gastric impaction. Lesions were most frequently identified in the digestive (58/61), integumentary, visual, musculoskeletal, cardiovascular, urinary, hematopoietic systems, and less frequently in the nervous and endocrine systems. Cachexia, presented based on the observation of muscle atrophy, enophthalmos and plastron concavity, was observed in 49.2% of the turtles (30/61). The results of this study provide information on pathological conditions that may be of clinical relevance for the rehabilitation of animals and their reintroduction to their natural habitat, reducing the impacts of these factors on the conservation of these species and the health of the marine ecosystem.

## Introduction

Sea turtles play an important role in the nutrient cycle and community structure of their feeding grounds. In the wild, under suitable conditions, turtle populations in feeding grounds are high, making them important predators and grazers. They export nutrients from their feeding grounds, bringing health and balance to the marine environment. [1]. There are significant records of sea turtles stranded on Brazilian beaches, occurring in several nesting, feeding, or migration areas [2–4]. Four of the five species of sea turtles that occur in Brazil (*Caretta caretta, Eretmochelys imbricata, Lepidochelys olivacea, and Dermochelys coriacea*) are currently under threat of extinction [5–9]. However, *Chelonia mydas* has had its status changed to “Near Threatened” since 2022, being removed from the Official List of Threatened Species of Brazilian Fauna [9–10]. Despite this improvement in its condition, its current situation still requires conservation actions due to the threats to which these animals are exposed [10].

In general, information about what happens to turtle cadavers in the marine environment is still not very representative, and it is difficult to determine the time of death of the animal. It is estimated that strandings account for around 10–20% of the total mortality of animals on the high seas, due to the variable probability of a cadaver reaching the coast [11]. Factors such as wind direction, sea currents, and the action of predators, among others, can prevent cadavers from reaching the beaches. And when this happens, the first step is to identify the cause of death, especially if there is some anthropogenic relationship involved [12].

Determining the cause of death for stranding becomes challenging due to the possibility of two or more factors being involved in this cause, or due to the degree of decomposition of the corpse. Cases suggestive of drowning represent an additional challenge, since the necroscopic findings are unstable and disappear quickly [13]. Situations in which the stranded animal has a good body condition score or is in an active reproductive phase suggest death or acute injury, since chronic conditions would lead this individual to a state of inappetence, thinness, or cachexia, and the same would possibly be unfit for reproduction [13]. In addition to infectious diseases [14], fishing activity, improper waste disposal, indiscriminate use of pollutants, habitat modification, and disorderly coastal occupation are some of the main causes of sea turtle strandings on the Brazilian coast. [15,16].

In recent years, research has been carried out to understand the changes/injuries that affect sea turtles in different parts of the world, with necropsy being one of the main diagnostic tools for determining the diseases affecting this population [17–23]. This diagnostic tool allows us to investigate and identify the cause of the animal’s death, as well as to provide important information regarding biological aspects and pathological findings that will contribute to the management and conservation of these species [17–20,22]. However, even considering the data already presented in the literature, the development of research aimed at understanding the regional factors that affect the epidemiology of certain diseases is necessary and pertinent, supporting the development of strategies and interventions directed towards this end [24–26].

Therefore, systematic monitoring of sea turtle strandings will provide important biological information for the management and conservation of these animals, in addition to allowing the analysis of historical data series on the mortality of different species [11]. Thus, the objective of this study was to identify the cause of death in green turtles (*Chelonia mydas*) on the northern coast of Bahia, and its impact on sea turtle conservation.

## Materials and methods

### Area of study

The study was developed with animals that stranded on the northern coast of Bahia, mostly in the coastal area of Praia do Forte, which has 12 kilometers of coastline, with latitude: 12.574743 and longitude: 38.0044715, where the base of the Fundação Projeto Tamar is located.

### Ethical considerations

The protocols of this research were approved by the Ethics and Animal Welfare Committee of the School of Veterinary Medicine and Animal Science of the Federal University of Bahia (protocol no. 90/2018), are in accordance with the Biodiversity Information Authorization System of the Brazilian Ministry of the Environment – SISBIO (processes no. 64518−1 and no. 64518−3) and certified by the National System for the Management of Genetic Heritage and Associated Traditional Knowledge – SISGEN (registration no. A0F349F).

### Research execution stages and locations

The research was developed in an interinstitutional partnership (Federal University of Bahia – UFBA, in the Veterinary Pathology Laboratory – LPV/ Federal University of Recôncavo da Bahia – UFRB, in the Parasitology and Parasitic Diseases Laboratory – LPDPV/ University of São Paulo – USP, in the Comparative Pathology of Wild Animals Laboratory – LAPCOM/ Fundação Projeto Tamar), in two stages: a) necroscopic examination of the turtles, at LPV-UFBA; b) processing and analysis of the samples collected for histopathological and histochemical examinations, respectively.

### Animals and sample collection

Specimens of free-living *Chelonia mydas* that washed ashore between February 2018 and September 2019 on the beaches of the northern coast of Bahia, in the monitoring area of the Fundação Projeto Tamar, found dead, or that died during treatment and rehabilitation at the Fundação Projeto Tamar, were necropsied for this study. Animals that lost their quality of life, without responding to supportive treatment, were euthanized to minimize their suffering. The protocol for this procedure consisted of manual restraint and intravenous injection of propofol (12 mg/kg) into the dorsal cervical sinus, monitoring for loss of the palpebral reflex. The animals were then placed in dorsal recumbency to monitor for loss of sensation and cloacal reflex. After evaluating these parameters, potassium chloride (2 mEq/kg) was administered intracardiac through the plastron to stop cardiac movement. The frozen animals were kept in the freezer (−20°C), and upon arrival at LPV-UFBA, they were kept in the cold chamber (4°C) so that thawing could occur in a temperature-controlled environment. As for fresh animals, necropsy was performed as soon as they arrived at the laboratory.

The classification of the cadavers’ condition at necropsy (CCN) was performed based on an adaptation of the “Protocol of Conduct for Stranding of Aquatic Mammals from the Rede de Encalhe de Mamíferos Aquáticos do Nordeste (REMANE)” [27], which in turn was based on the classification established by Geraci and Lounsbury [28]. Thus, concerning the state of conservation, the groups were classified as: fresh cadaver (recent death) (Class 2); cadaver with identifiable and intact internal organs (Class 3); cadaver in advanced decomposition (unidentifiable internal organs) (Class 4) and mummified cadaver or skeleton (Class 5). To carry out the first stage, a specific necropsy data sheet for sea turtles was prepared based on data collected from the form used at Fundação Projeto Tamar and anatomical locations according to “The Anatomy of Sea Turtles” by Wyneken [29].

### Determination of cause of death

The causes of stranding were classified according to the nature of death into natural threats – NT (pathological processes of various origins such as parasitic, bacterial, fungal, metabolic), anthropogenic interaction – AI (any human action, regardless of the type of interaction with fishing or solid waste) or inconclusive – IC (when it was not possible to identify the origin of the cause of death) and the most important pathological process associated with the death of the animal. To identify animals with possible interaction with fishing, information in the historical form sent by the Fundação Projeto Tamar was collected, which indicated this information, followed by descriptions of fishing artifacts or lesions noticed upon external examination, such as lesions on limbs, neck, head, carapace, and plastron. Additionally, a good body score of animals found dead, but apparently healthy, and with findings of fluid in the lungs and/or gastrointestinal tract in the necroscopic examination, with no other evidence that corroborated an acute death, were considered to determine whether the animals were likely to have undergone anthropic interaction.

### Histopathological processing and analysis

For histopathological analysis, all samples were processed using the routine histological technique of paraffin embedding [30]. Sections of 4 μm were made and stained with hematoxylin-eosin (HE) and special stains: Gram, Ziehl Neelsen (ZN), Periodic Acid Schiffer (PAS), Giemsa, and Masson’s Trichrome. At the end of this stage, the histological sections were analyzed under an optical microscope, with a camera attached to capture images (Leica® Microscope). The cases were divided and categorized into three groups according to the CCN, and the description of the analyses was performed according to the systems that presented alterations. To perform this classification, all necropsy and photographic records available for the animals were reviewed, and the data were compiled in Excel® spreadsheets. All captured photomicrographs were standardized in the ImageJ software.

## Results

Sixty-one animals (two adults and 59 juveniles) of the species *C. mydas* were necropsied. 75.4% (46/61) were females, including the adults, and 24.5% (15/61) were males. In the history of strandings of these turtles, 13 animals stranded alive and were sent for rehabilitation at the Fundação Projeto Tamar, where they died, and 48 turtles were found dead on the beaches of the northern coast of Bahia. Of the 13 turtles treated while still alive, 11 were juveniles (five females and six males), and two were adults. Eight had poor body condition scores, two had average scores, and two had good scores. Of these, two had a history of interaction with fishing. The euthanized turtles were female juveniles, both cachectic and without obvious signs of interaction with fishing. However, one had solid waste (plastic) in its gastrointestinal tract, causing fecal impaction, intestinal synechiae, severe hydrocoelom, hepatomegaly, splenomegaly, and bone fragility. The other euthanized turtle had pulmonary edema and congestion, nephritis, and renal congestion. In both cases, euthanasia was important to alleviate the animals’ suffering.

### Anatomopathological findings

Regarding the condition of the cadavers at necropsy, 22.9% (14/61) of the animals were considered class 2, of which 21.4% (3/14) were fresh animals and 78.6% (11/14) were frozen; 77% (47/61) were considered class 3. Epibionts (barnacles, leeches, and algae) were observed in 67.2% (41/61) of the animals, and the barnacles were mostly distributed in fins, carapace, plastron, cervical region, and head. It was possible to determine and classify the cause of death of the turtles in 96.7% (59/61) of the animals, with 77% (47/61) being NT, 19.7% (12/61) of the cases due to IA and 3.3% (2/61) were IC, as they were animals subjected to euthanasia (Table 1).

**Table 1:** Cause of death of *Chelonia mydas* stranded on the northern coast of Bahia.

Cause of death (Pathological processes)	Number of animals				
	Nature of death			Total	(%)
	NT	AI	IC		
Acute respiratory failure	23	4	–	27	44.3
Sepsis	12	5	–	17	27.9
Cachexia/ Malnutrition	8	2	–	10	16.4
Circulatory collapse due to thromboembolism	4	–	–	4	6.5
Gastric impaction	–	1	–	1	1.6
Euthanasia	–	–	2	2	3.3
Total	**47**	**12**	**2**	**61**	**100**

**NT –** natural threats; **AI –** anthropic interactions; **IC –** inconclusive.

Cachexia was diagnosed in 30/61 turtles, based on the association of the following characteristics: muscle and reserve fat atrophy, enophthalmos, and plastron concavity.

Of the necropsied animals, 13/61 were submitted to treatment while alive, at necropsy, nine presented free liquid in the coelomic cavity in varying quantities, in six animals, the hydrocoelom was scarce to moderate (100mL – 2L), and in three it was abundant (3,8 and 25L), with the largest volume being equal to 25 liters in an adult animal. Of the dead stranded animals, 14/61 had free fluid in the coelomic cavity, 12 had hydrocoelom ranging from scarce to moderate (50mL – 700mL), and hemocoelom was observed in 2 animals (200mL – 250mL); one animal had sepsis, and the other had severe parasitic infection.

Lesions were most frequently identified in the digestive (58/61), respiratory (55/61), integumentary (46/61), musculoskeletal (36/61), cardiovascular (30/61), urinary (27/61), and hematopoietic (14/61) systems. The other systems examined presented a lower frequency of lesions, including the nervous (06/61) and endocrine (04/61) systems. Tables 2 and 3 quantify the macroscopic and histopathological findings observed in the animals necropsied in this study.

**Table 2:** Macroscopic findings in *Chelonia mydas* stranded on the northern coast of Bahia, Brazil.

Macroscopic findings		N 61(%)
Digestive System		
Esophagus	**Esophagitis**	4 (6.5%)
Stomach	**Gastritis**	11 (18%)
	**Gastric impaction**	1 (1.6%)
Intestines	**Enteritis**	39 (63.9%)
	**Intestinal impaction**	8 (13.1%)
	**Megacolon**	1 (1.6%)
Liver	**Congestion**	13 (21.3%)
	**Hepatomegaly**	2 (3.3%)
	**Evidence of the lobular pattern**	6 (9.8%)
	**Hepatic lipidosis**	5 (8.2%)
	**Icterus and liver fibrosis**	1 (1.6%)
	**Hepatitis**	5 (8.2%)
Respiratory system		
Lungs	**Edema**	50 (82%)
	**Congestion**	9 (14.7%)
	**Hemorrhages**	9 (14.7%)
	**Atelectasis**	2 (3.3%)
	**Emphysema**	6 (9.8%)
	**Granulomas**	3 (4.9%)
	**Fibroma**	2 (3.3%)
Cardiovascular System		
Pericardium	**Hydropericardium**	20 (32.8%)
	**Fibrinous pericarditis**	2 (3.3%)
Blood vessels	**Endarteritis**	2 (3.3%)
	**Granulomatous arteritis**	3 (4.9%)
Heart	**Fibrinous epicarditis**	2 (3.3%)
	**Myocarditis**	3 (4.9%)
	**Concentric hypertrophy**	1 (1.6%)
Urogenital system		
Kidneys	**Congestion**	12 (19.7%)
	**Edema**	2 (3.3%)
	**Fibrosis**	2 (3.3%)
	**Fibroma**	2 (3.3%)
Urinary bladder	**Cystitis**	2 (3.3%)
	**Hematuria**	1 (1.6%)
	**Urolithiasis**	1 (1.6%)
Integumentary System		
Skin	**Ulcerative dermatitis**	3 (4.9%)
	**Fibropapillomatosis**	34 (55.7%)

**Table 3:** Microscopic findings in *Chelonia mydas* stranded on the northern coast of Bahia, Brazil.

Microscopic findings		N 61(%)
Digestive System		
Esophagus	**Parasitic ulcerative esophagitis**	3 (4.9%)
Stomach	**Erosive Gastritis**	2 (3.3%)
	**Ulcerative gastritis**	1 (1.6%)
	**Gastritis with parasitic granulomatous vasculitis**	13 (21.3%)
	**Fibroma**	1 (1.6%)
Intestines	**Parasitic granulomatous enteritis and vasculitis**	3 (4.9%)
	**Segmental fibrinonecrotic enteritis**	3 (4.9%)
	**Segmental necrotic enteritis**	2 (3.3%)
	**Severe segmental hemorrhagic fibrinonecrotic enteritis with lymphangiectasia associated with coccidian infection**	1 (1.6%)
	**Bacterial enteritis**	1 (1.6%)
	**Fungal enteritis**	2 (3.3%)
Liver	**Heterophilic granulomatous hepatitis**	20 (32.7%)
	**Parasitic granulomatous hepatitis**	6 (9.8%)
	**Fungal hepatitis**	2 (3.3%)
	**Bacterial hepatitis**	1 (1.6%)
	**Protozoal hepatitis**	1 (1.6%)
	**Fibrosis**	1 (1.6%)
Respiratory system		
Lungs	**Granulomatous pneumonia**	36 (59%)
	**Exudative bronchopneumonia**	12 (19.7%)
	**Fibrosis**	1 (1.6%)
	**Visceral fibroma**	2 (3.3%)
Cardiovascular System		
Pericardium	**Heterophilic pericarditis**	1 (1.6%)
	**Granulomatous and parasitic pericarditis**	1 (1.6%)
Blood vessels	**Parasitic granulomatous arteritis**	6 (9.8%)
	**Proliferating endarteritis**	6 (9.8%)
Heart	**Fibrinous epicarditis**	2 (3.3%)
	**Heterophilic myocarditis**	7 (11.5%)
	**Fibrinoparasitic myocarditis**	5 (8.2%)
	**Myocarditis and parasitic granulomatous vasculitis**	3 (4.9%)
Urogenital system		
Kidneys	**Heterophilic nephritis and tubular necrosis**	1(1.6%)
	**Lymphocytic nephritis**	2 (3.3%)
	**Fungal granulomatous nephritis**	1 (1.6%)
	**Nephritis with parasitic granulomatous vasculitis**	22 (36.1%)
	**Fibrosis**	2 (3.3%)
	**Visceral fibroma**	2 (3.3%)
Urinary bladder	**Cystitis**	3 (4.9%)
	**Serositis with granulomatous vasculitis**	2 (3.3%)
Intergumentary system		
Skin	**Ulcerative dermatitis**	3 (4.9%)
	**Fibropapillomatosis**	34 (55.7%)
	**Fibropapillomatosis associated with spirorchids**	8 (13.1%)

### Digestive system

The lesions observed in the esophagus were ulcerative esophagitis and, microscopically, parasite eggs were observed. Lesions in the stomach characterized as mucous, hemorrhagic, parasitic, granulomatous, erosive and/or ulcerative gastritis were identified. Some lesions affected more than one segment, and gastroesophagitis and gastroenteritis were observed. In the histopathological evaluation, the most frequent lesion was gastritis with parasitic granulomatous vasculitis, associated with the presence of parasite eggs. Other alterations included erosive/ulcerative gastritis and one case of visceral fibroma.

**Fig 1:**
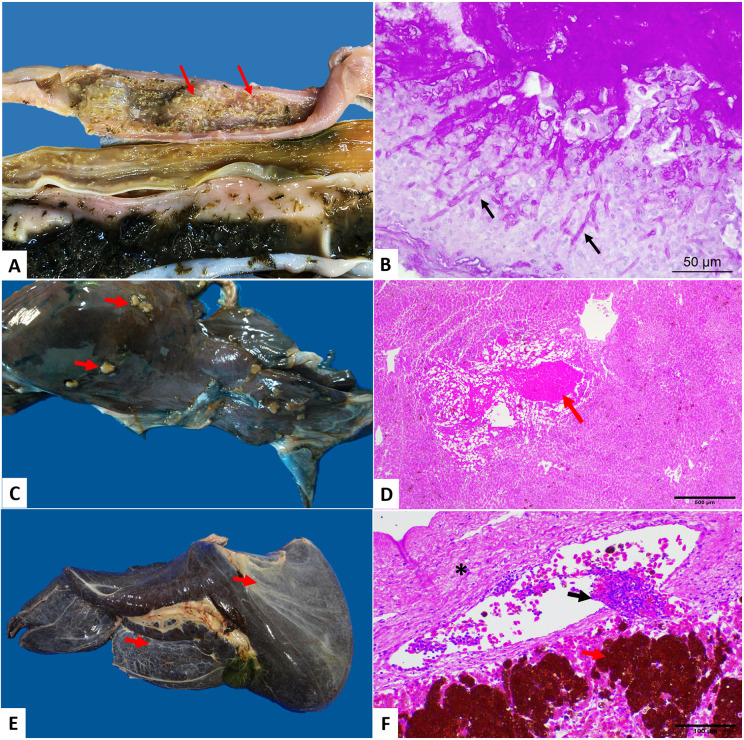
Morphological findings in the digestive system of *Chelonia mydas.* (A-B) Fungal fibrinonecrotic enteritis. (B) Photomicrograph showing intralesional hyphae (black arrows). Obj. 40x. (C) Multifocal hepatic granulomas (arrows). (D) Granulomatous hepatitis (note the necrotic center – arrow). Obj. 4x. (E) Hepatic fibrosis (arrows). (F) Inflammatory reaction in the liver parenchyma (arrows). Note clusters of melanomacrophages in the liver parenchyma, fibrosis of the capsule (asterisk). Obj. 20x. PAS (B) and HE staining.

Enteritis was the most frequent lesion (39/61), with most cases being parasitic. Other types observed were catarrhal, hemorrhagic, ulcerative and necrotizing enteritis. Cases of intestinal compaction were observed, seven of which were associated with the presence of solid waste and one animal had a megacolon measuring 23 x 22 cm. However, intestinal dilation with compaction was not associated with the presence of anthropogenic waste, but there was a discreet parasitic infection. Microscopically, the relevant findings observed were parasitic granulomatous enteritis and vasculitis caused by parasites of the Spirorchiidae family, segmental fibrinonecrotic enteritis, segmental necrotic enteritis, severe segmental hemorrhagic fibrinonecrotic enteritis with lymphangiectasia, associated with infection by the coccidian *Caryospora* sp. (Léger, 1904) (Apicomplexa: Eimeriidae), with a positive result on PAS stain. One of the above cases was positive on Gram stain for gram-negative bacteria; of the three cases of fibrinonecrotic enteritis, one was of fungal origin with visualization of hyphae on PAS stain (Figs 1A and 1B).

Hepatic congestion was the most common pathological process observed in the liver of the animals, with two turtles presenting congestive hepatomegaly, as well as evidence of the lobular pattern and hepatic lipidosis. Hepatitis varied from granulomatous to abscessive, characterized by small yellowish-whitish abscesses, randomly distributed throughout the liver (Figs 1C and 1D). One of the animals presented mild icterus associated with hepatic fibrosis (Figs 1E and 1F). The main liver histopathological diagnosis was heterophilic granulomatous hepatitis, sometimes associated with parasitic or fungal infection. One of the cases tested positive for acid-fast bacteria (AFB) in the ZN stain. Cases of parasitic granulomatous hepatitis and vasculitis were observed, in addition to other changes observed, such as hemorrhage, fibrosis with positivity to Masson’s Trichrome, and marked presence of melanomacrophages.

### Respiratory system

**Fig 2:**
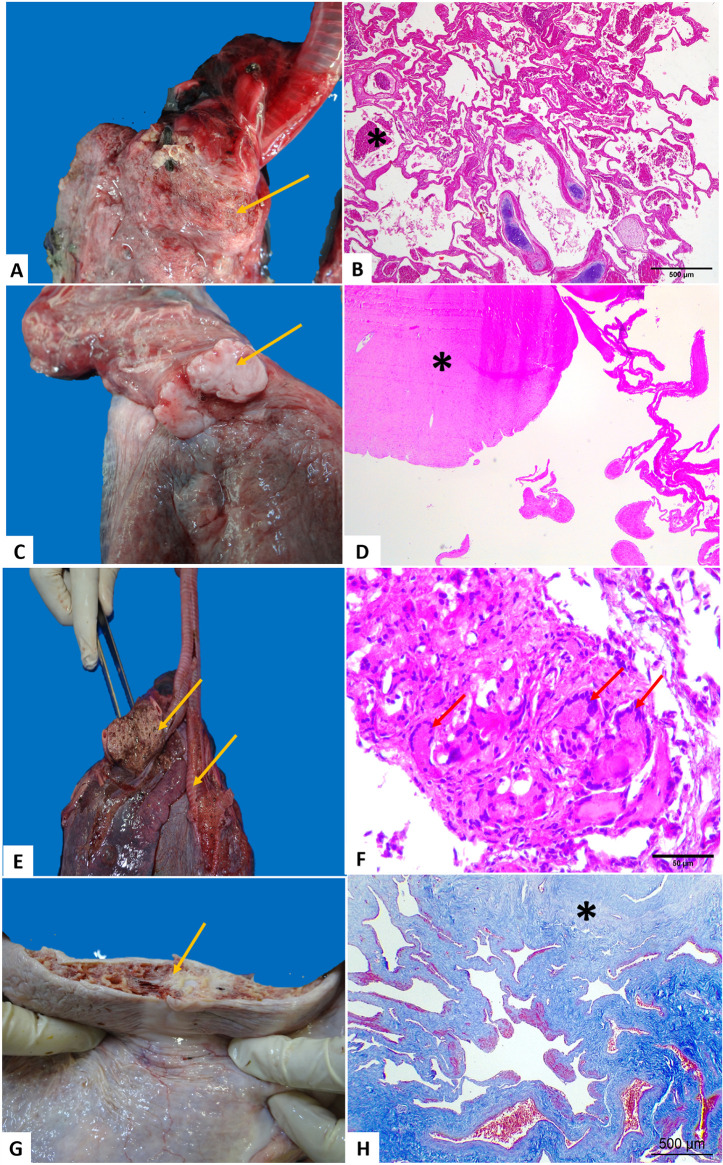
Anatomopathological findings in the lungs of *Chelonia mydas.* (A-B) Exudative pneumonia. (B) Photomicrograph of lung parenchyma with air spaces filled with heterophilic inflammatory infiltrate, obj. 4x. (C) and (D) Pulmonary visceral fibroma, in (D) photomicrograph obj. 4x. (E) Granuloma in the cranial region of the lung parenchyma (arrow). (F) Granulomatous reaction in the lung parenchyma; note the giant, multinucleated cells (arrows). (G) Fibrosis in the lung parenchyma (arrow). Note the cut surface of the lesion (arrow). (H) Pulmonary fibrosis; note the extensive areas of connective tissue stained blue by Masson’s Trichrome. Obj. 4x. Figures B, D and F, HE staining.

Pulmonary edema was the most frequently observed alteration in the respiratory system, followed by congestion, hemorrhage, atelectasis, and emphysema. Exudative bronchopneumonia was characterized by the presence of numerous heterophils in the central lumen of the bronchi, associated or not with bacterial colonies. Granulomatous pneumonia was characterized by the presence of well-defined granulomas containing central areas of eosinophilic necrotic debris associated with heterophils and macrophages. Multinucleated giant cells surrounding necrotic debris were observed in some granulomas, mostly associated with parasitic eggs, in addition to fibrosis, and two cases of visceral fibroma in turtles with fibropapillomatosis. All granulomas were negative for AFB in the ZN and PAS reaction. In most cases, the pneumonic lesions were bilateral (Fig 2).

### Cardiovascular system

**Fig 3:**
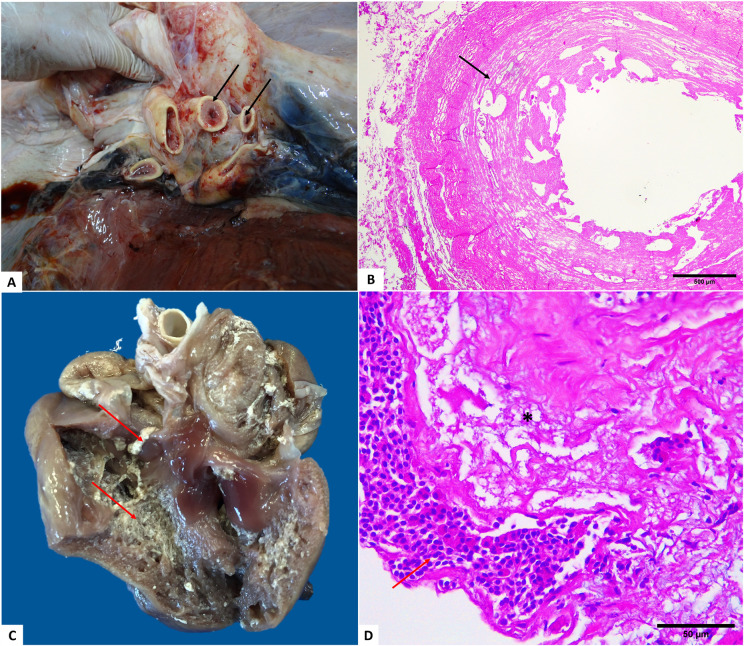
Macroscopic findings in the cardiovascular system of *Chelonia mydas.* **Morphological lesions in the cardiovascular system.** (A) Endarteritis in the large vessels of the heart (arrows). (B) Proliferative endarteritis in the aorta; proliferation of the media and intima layers (arrow) of the vessel, reducing the arterial lumen. (C) Fibrinous myocarditis (arrows show fibrin filaments). (D) Fibrinous myocarditis; fibrin filaments (asterisk) associated with mixed inflammatory infiltrate (arrow). Obj. 40x. B. Obj. 4x. HE staining.

The most commonly identified macroscopic alteration was hydropericardium, followed by endarteritis associated or not with thrombosis; granulomatous arteritis, granulomatous myocarditis, fibrinous pericarditis, fibrinous epicarditis, and concentric hypertrophy. In the histopathological analysis, the main alterations observed were heterophilic myocarditis, parasitic granulomatous arteritis and proliferating endarteritis, fibrinoparasitic myocarditis, parasitic granulomatous myocarditis and vasculitis, in addition to one case of cardiac hypertrophy (Fig 3).

### Urogenital system

**Fig 4:**
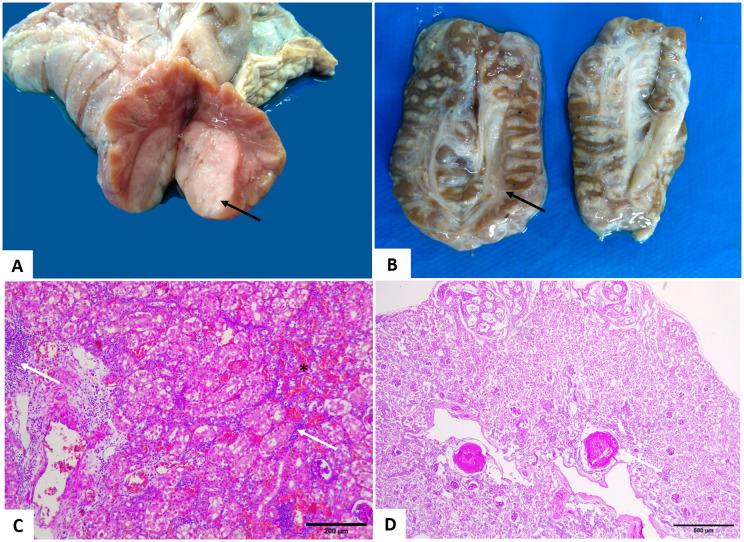
Anatomopathological findings in the kidney of *Chelonia mydas.* (A) Visceral fibroma in the caudal pole of the kidney, cut surface – note the whitish coloration, compact, delimited, and expansive growth (arrow). (B) Renal fibrosis. Note bundles of connective tissue compressing the renal parenchyma. (C) Interstitial nephritis. Note the cluster of inflammatory cells (arrows) and congestion (asterisks). (D) Granulomatous nephritis. Heterophilic granulomas (arrow) Obj. 10x; 4x. HE staining.

Renal congestion was identified in 12/61 turtles that presented with some lesion in this system. In some cases, there were spots or blackened areas on the capsular and cut surfaces, which in the histopathological evaluation were characterized as nephritis associated with parasitic granulomatous vasculitis. Subcapsular edema, renal fibrosis, and visceral fibroma were observed in two turtles with fibropapillomatosis. Hematuria and hemorrhagic cystitis were diagnosed in the bladder, as well as vesical urolithiasis in one juvenile female animal, and the presence of the specimen *Plesiochorus* sp. (Looss, 1901) (Trematoda: Gorgoderidae). Nephritis with parasitic granulomatous vasculitis associated with parasite eggs was the most frequently identified alteration in the histopathological examination, followed by heterophilic nephritis, tubular necrosis, lymphocytic nephritis, and renal fibrosis. One of the cases of granulomatous nephritis was positive in the PAS stain for fungal lesions. Congestion and hemorrhage were also observed. The histopathological examination of the bladder showed cystitis and serositis with granulomatous vasculitis (Fig 4).

### Hematopoietic, nervous, and endocrine systems

Splenitis with parasitic granulomatous vasculitis (23/61), splenic hemorrhage (5/61), fibrinoid necrosis of splenic arterioles and fibrosis (1/61) were the lesions observed in the hematopoietic system. In some cases, melanomacrophages were diffusely distributed. The case of splenic fibrosis was confirmed by Masson’s Trichrome staining. In the central nervous system, histopathological findings of meningoencephalitis (6/61), meningitis (1/61), and encephalitis (2/61) were associated with granulomatous and parasitic vasculitis. Vasogenic edema was seen in one case. In the endocrine system, hypertrophy and hyperplasia of pancreatic ducts, capsular fibrosis, and parasitic granulomatous vasculitis (3/61) were identified, with one case of necrotizing cystic thyroiditis and thyroiditis with parasitic granulomatous vasculitis (3/61). Adrenalitis (3/61) was also observed.

### Integumentary system

**Fig 5:**
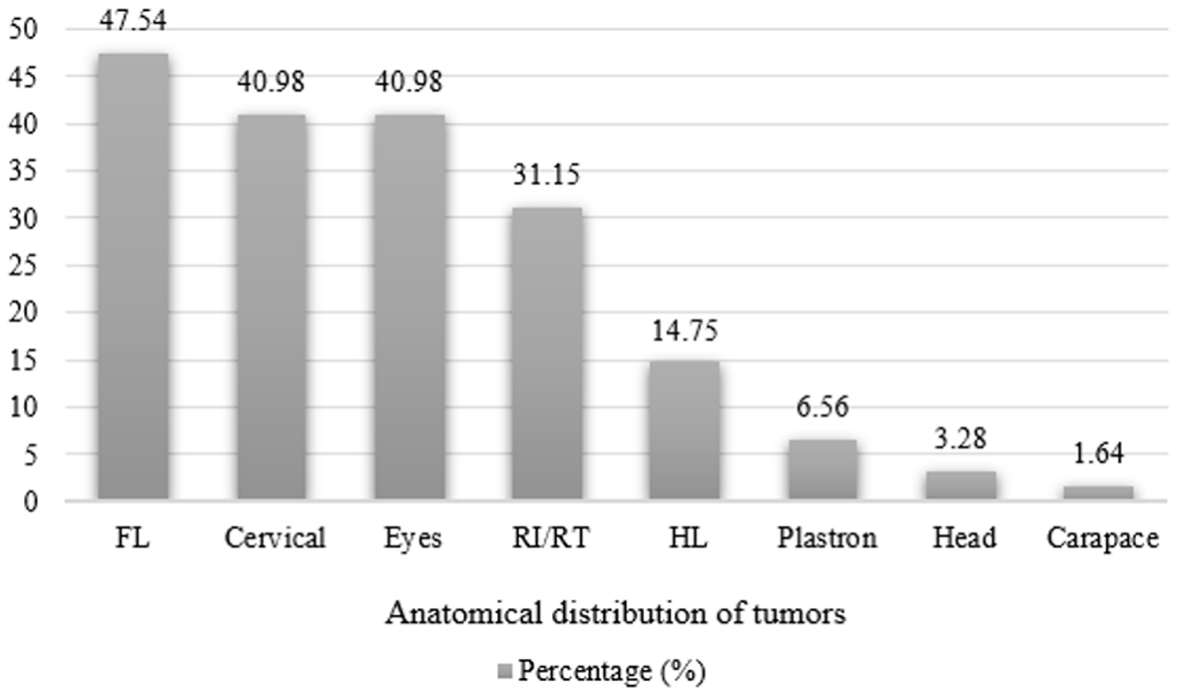
Occurrence (%) of cutaneous fibropapillomas in *Chelonia mydas* according to anatomical location. **FL –** forelimbs; **RI/RT –** inguinal region/tail.; **HL –** hindlimbs.

**Fig 6:**
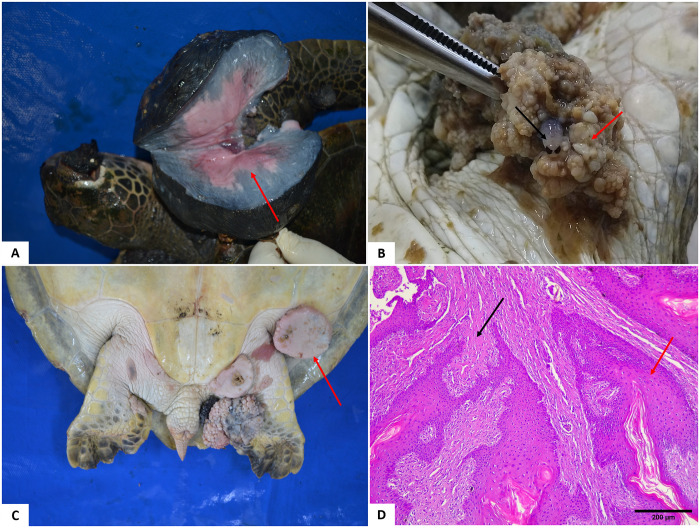
Cutaneous fibropapillomas in *Chelonia mydas.* (A-C) Distribution of cutaneous fibropapillomas. (A) Right forelimb and eye – tumor cut surface. (B) A specimen of *Euridice affinis* (black arrow) inside the tumor (red arrow). (C) Fibropapillomas in the inguinal region (arrow). (D) Photomicrograph of fibropapillomas. Proliferation of epithelial cells of the spinous layer is associated with reactivity and proliferation of fibroblasts (black arrow), with a papillary appearance (red arrows). Note the hyperkeratosis. Obj. 10x. HE staining.

Thirty-four turtles presented non-traumatic lesions, ulcerative dermatitis, and proliferative lesions resulting from fibropapillomatosis (FP). Ulcerative dermatitis was characterized by the presence of ulcers with necrosis of the epidermis and dermis, in addition to the presence of granulocytes and infiltrating bacteria, associated with the underlying layer of multinucleated giant cells. Of the animals evaluated, 44.3% (27/61) did not present skin lesions. Fig 5. shows the distribution of FP according to the anatomical region. The number of tumors in the different anatomical regions does not mean that several turtles were affected; in some cases, a single individual had tumors in all these locations (Fig 6). In two juvenile animals, both female, some tumors had leeches, and in one juvenile female turtle, there was an Isopod specimen inside the tumor (Fig 6B). Microscopically, there were parasite eggs in the tumors of eight turtles.

### Anthropic interaction

Regarding anthropogenic interactions, a total of 26/61 turtles had some type of interaction, of which 11/26 were due to interaction with fishing, 8/26 due to the presence of anthropogenic waste, and 7/26 had both interaction with fishing and the presence of anthropogenic waste.

### Identification of anthropogenic waste and associated injuries

**Fig 7:**
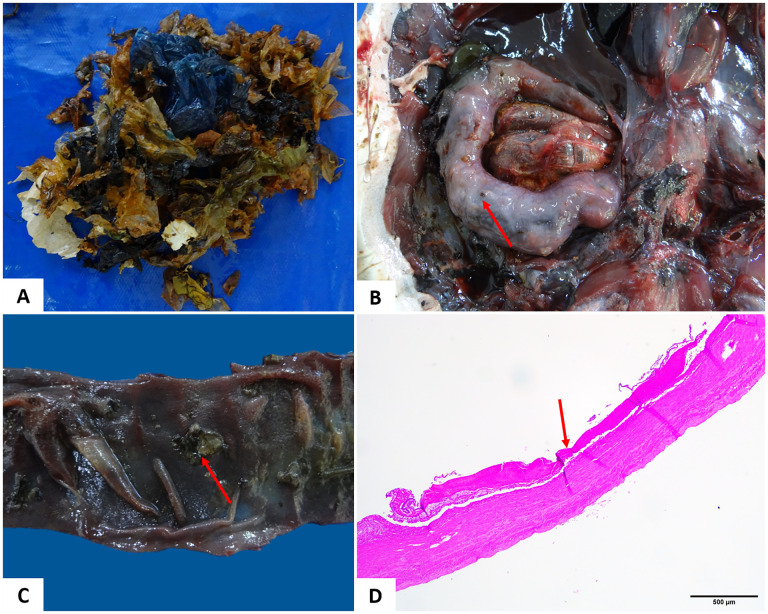
Anthropogenic debris and associated lesions identified in the intestine of *Chelonia mydas* from the northern coast of Bahia. (A) Fine plastics. (B) Intestinal compaction. (C) Intestinal segment with ulcerative lesions (arrow). (D) Photomicrograph of ulcerative enteritis (arrow). Obj. 4x. HE staining.

Overall, anthropogenic waste was observed in 15/61 sea turtles, and in eight of them, these materials were directly related to the cause of death. The category of material with the highest identification of types was plastic, both industrial and domestic (Fig 7A). Fine plastics were the most frequently observed solid waste (14/15), followed by line-type waste (fishing line and nylon thread). Both types were observed in 80% (12/15) of the turtles analyzed. Other types of residues were observed in ten of the affected turtles. Regarding location, in at least one of the 15 animals, some residue was observed in the esophagus, stomach, or intestine, although the segments of the intestinal loops were the most frequent region. Of the 15 sea turtles identified, ten presented some lesion in the GI tract associated with the presence of residues, four had alterations related to hemodynamic disorders such as edema of the stomach and/or intestine wall, sometimes with parasitic infection. Only one animal did not present lesion in the GI tract. Lesions such as hemorrhagic gastritis, erosive gastritis, ulcerative gastroenteritis, intestinal impaction, fibrinous ulcerative enteritis, perforating ulcerative enteritis, necrotizing enteritis and synechiae of intestinal loops were observed. In the coelomic cavity of 50% of the animals, there was a case of coelomitis, of which 20% (3/15) were of the fibrinous type; 30% (4/15) of the animals presented hydrocoelom and in the remaining animals there was no change in the cavity (Fig 7B-D). The general condition of all animals included cachexia, anemia and pallor of viscera; 80% (12/15) turtles were females and 20% (3/15) were males.

### Identification of suggestive injuries associated with interaction with fishing

**Fig 8:**
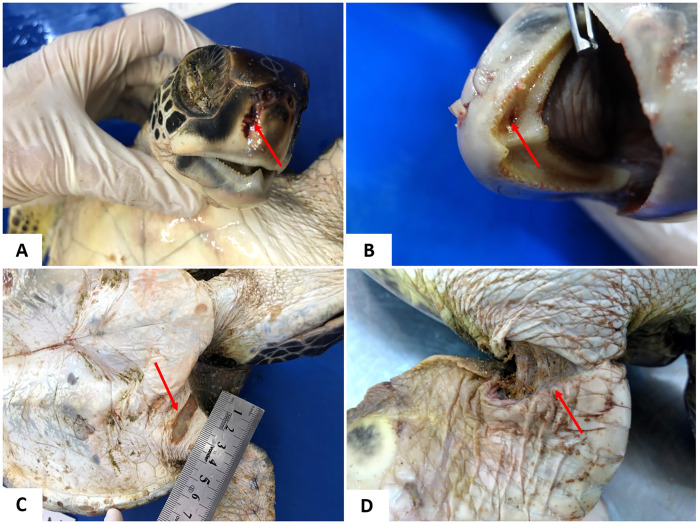
External injuries suggestive of interaction with fishing in *Chelonia mydas* stranded on the northern coast of Bahia. (A) Fracture and hemorrhage in the rhamphotheca region, close to the right nostril (arrow). (B) Perforating injury in the oral cavity. (C-D) Injuries to the forelimb.

A total of 18/61 sea turtles presented lesions suggestive of interaction with fishing (Fig 8); however, only four of these animals presented alterations of this origin that were related to death. These sea turtles presented acute respiratory failure, suggestive of drowning due to the presence of circulatory collapse characterized by hemorrhage, pulmonary congestion and edema, hydropericardium, cardiac hemorrhage, and renal congestion. However, because these animals were subjected to freezing, confirmation of the findings was impaired. One of these turtles (1/4) presented extensive hematoma and hemorrhage in the periosteum, unilateral pulmonary atelectasis, in addition to pulmonary edema and emphysema, multiple lesions in the right marginal shields, and lateral shields. This was one of the live stranded turtles submitted to treatment, presenting a poor body score. All turtles were juveniles, 50% (9/18) females and 50% (9/18) males. The individuals presented variable body condition, with 33.3% (6/18) classified as good, 22.2% (4/18) as average, and 44.4% (8/18) as poor. Lesions in the integumentary and musculoskeletal systems are shown in Table 4.

**Table 4:** Lesions in the integumentary and musculoskeletal systems in *Chelonia mydas* stranded on the northern coast of Bahia N = 18.

Region	Injury	N.	%
Head	Rhamphotheca fracture	2	11.1%
Carapace	Fracture in marginal shields	3	16.6%
	Fracture of lateral shields	3	16.6%
	Bone bleeding	1	5.5%
	Fracture of central shields	1	5.5%
Plastron	Fractures	2	11.1%
Forelimb	Skin laceration	2	11.1%
	Fractures	5	27.7%
	Hematoma	2	11.1%

## Discussion

This study was conducted with 61 turtles of the species *Chelonia mydas* stranded on the northern coast of the state of Bahia, with the capture bases for these animals located mostly in Praia do Forte, followed by Arembepe and Sauípe. All these animals were directed to the base of the Fundação Projeto Tamar located in Praia do Forte. Similar to other studies, most of the animals were females in the juvenile age group [17,18,22].

A survey of data on sea turtle strandings in the areas covered by the Fundação Projeto Tamar in the northeast region from 2010 to 2012 revealed 5,726 events, with a higher frequency of individuals of the species *C. mydas* [4,036 (70.5%)], followed by 1,181 (20.62%) *L. olivacea*, 276 (4.82%) *E. imbricata*, 88 (1.53%) *C. caretta*, and, to a lesser extent, *D. coriacea* [4 (0.07%)], of which 141 (2.46%) specimens were not identified. Of the species identified, 3,653 (65.40%) were juvenile *C. mydas*, indicating the northeastern coast as a feeding area for young individuals of this species [6].

*Post-mortem* examinations of sea turtles have limitations, especially with histopathological evaluation due to the autolysis process [31–33]. However, in a study carried out with 93 turtles, it was not possible to perform histopathological examination in only seven individuals [18]. In the present study, there was a limitation in performing the diagnosis due to the decomposition of the cadavers; in these cases, although the conditions were not ideal, they did not prevent the histopathological diagnosis of these animals [24].

### Anatomopathological findings

The number of animals with a large amount of free fluid in the coelomic cavity was lower than that observed in the study carried out by Cruz-Uchoa [22], who reported this alteration in 59.18% (29/49) of the animals studied. The presence of free fluid may be associated with the administration of Ringer’s solution, via intracoelomic route, a clinical procedure for hydrating the patient when admitted to the rehabilitation center [34]. The cases of hydrocoelom observed in the present study are not related to intracoelomic fluid therapy, since the route commonly used in the rehabilitation center of the Fundação Projeto Tamar, Praia do Forte base, is intravenous and, less frequently, subcutaneous. Cavitary edema in these animals may be related to hemodynamic and nutritional disorders that they presented, such as malnutrition, cachexia and starvation caused by chronic, parasitic and infectious diseases, and by the ingestion of anthropogenic waste, conditions that lead the patient to anemia and hypoproteinemia, which are the result of increased vascular permeability, intravascular hydrostatic pressure and decreased intravascular osmotic pressure [35,36].

Lung lesions, observed as the most prevalent among the systems, have also been reported in other studies [18,19,22]. Silva et al. [37] analyzed the lungs of 29 individuals of the species *Eretmochelys imbricata* that had washed ashore on beaches in Espírito Santo; all animals presented lung lesions and were associated with specimens with poor body condition. In the present study, of the 55 animals that presented alterations in the respiratory system, 50.8% (31/61) presented poor body condition. In the case of these animals, lung lesions may be primary and predisposed to weight loss, or these lesions may be secondary to other diseases that occur with weight loss.

Lesions in the hepatobiliary system are frequently observed in sea turtles [17]. Several factors can trigger inflammatory processes in the liver, which may be of infectious origin caused by bacteria, viruses, or protozoa [38,39]. Granulomatous hepatitis was characterized macroscopically by the presence of granulomas of varying sizes randomly distributed throughout the parenchyma. Microscopy showed a lesion delimited by inflammatory cells, especially multinucleated macrophages surrounding an eosinophilic necrotic area, some with fungal hyphae, bacterial colonies, and/or parasite eggs [18]. In the present study, heterophilic granulomatous hepatitis was observed in some animals, and although tests for isolation of fungi and bacteria were not performed, some samples were positive in Gram staining for both Gram-positive and Gram-negative bacteria. It is known that these bacteria are associated with the development of granulomas in several organs, including the liver [18].

The muscular atrophy frequently observed in the animals in the present study is probably related to gastrointestinal disorders and concomitant immunosuppression caused by fibropapillomatosis, or even by weakness resulting from traumatic injuries of anthropic origin. The pallor, often observed, is related to anemia and cachexia, also identified in this study and corroborated by other authors [31].

Fibropapillomatosis is a disease that affects green turtles worldwide. It is characterized by the presence of benign neoplasms with variable histological types, such as papilloma, fibropapillomas, and fibromas, but fibrosarcomas and myxosarcomas have also been observed. It is believed to have a multifactorial origin, and its primary cause is *Chelonid alphaherpesvirus 5*. (ChHV-5) [14,40–44]. More than 50% (34/61) of the turtles evaluated in the present study presented cutaneous fibropapillomatosis, some of which also presented the visceral form, with varied sizes and anatomical locations. Once infected, the development of these tumors may be associated with genetic and environmental factors with a direct influence on the animal’s immune status, since stress conditions and immune deficits favor the development of FP [43,45]. Furthermore, it was observed that the presence of anthropogenic and parasitic factors may act as a predisposing element to FP, since intralesional leeches and parasites were identified in tumor samples, confirmed by histopathological analysis. These findings suggest that coinfection and anthropogenic interference may play a relevant role in the pathogenesis and severity of the lesions [43,46].

In general, the cutaneous tumors observed in this study were sessile, pedunculated, non-lobed to multilobed, smooth to irregular. According to color, the formations were pinkish to blackish, and some were ulcerated, corroborating the data of other authors [22,47,48]. The internal tumors were firm, whitish, and located in the kidneys and lungs, histologically characterized as fibromas [14]. Although it was not found in the present study, it is known that other internal organs can also be affected, such as the heart, intestines [14,47,49,50], muscle, spleen, and liver [47]. A study carried out by Dutra et al. [51] on turtles from the São Paulo region – Brazil, demonstrated the presence of visceral fibropapillomatosis in animals with cutaneous fibropapilloma. The anatomical region with the highest incidence of these tumors was the flippers, especially the front ones, followed by the cervical region, eyes, and inguinal region. The greatest anatomical distribution was in the frontal region (eyes, flippers, and neck), data that agrees with other studies conducted in Brazil [21,52–56]. Although previous studies indicate and corroborate sites with a higher incidence of fibropapillomas in turtles, no explanation or hypothesis for this predilection was observed in the available literature.

Six animals presented tumors between 4 and 10 centimeters and larger than 10 centimeters in the eye region, showing the severity and degree of impairment and impediment of the functions of these animals, such as adequate feeding. Tumors of this size were observed, especially in the front fins and neck, a situation that can also compromise the hydrodynamics and feeding of these animals and favor their weakness [14,21,56].

Traumatic skin lesions associated with secondary bacterial infection may be responsible for cases of sepsis that progress to death, and which could be associated with agents such as *Aeromonas hydrophila*, *Vibrio alginolyticus,* and *Staphylococcus* sp. [18,31]. Some suggestive sepsis conditions observed in this study were related to skin and carapace lesions due to trauma, in addition to cases of ulcerative dermatitis in the cervical region.

### Anthropogenic interaction

Knowledge about the ingestion of anthropogenic waste by marine animals is of fundamental importance, especially in endangered species such as sea turtles. Santos et al. [57] consider sea turtles as an iconic group of animals threatened by the ingestion of debris. These authors identified plastic as the most common type, followed by nylon/rope, which was also observed in the present study, since 93.3% (n = 14/15) and 80% (n = 12/15) of these types of debris, respectively, were identified. Plastic is the type of debris most often observed in turtles that ingest waste [18,58–61], and this data is also reported for other species, such as fish, seabirds, and sperm whales [62–65].

This is the second study on the ingestion of anthropogenic waste in *C. mydas* carried out on the northern coast of the state of Bahia. In a previous study, Macedo et al. [66] detected ingestion of high amounts of fishing-origin waste, as nylon threads and ropes, suggesting that the waste observed in the GI tract of these animals was ingested in the region where the research was carried out, given the characteristics of the waste found, since fishing and tourism activities are carried out in this region. Small amounts of waste are sufficient to cause intestinal obstruction and lead to the death of an animal [57,58].

In the present study, significant intestinal compaction was observed in one of the animals, which had only two nylon threads located in the esophagus, corroborating the cited literature. Intestinal obstruction caused by the presence of solid waste in marine animals can cause erosion, ulcers, or necrosis and indirectly affect lipid metabolism, prolonging intestinal transit time, worsening gas accumulation, and making floating difficult [67,68]. Cases of necrotizing enteritis due to intestinal intussusception secondary to fishing line ingestion were observed in a study carried out by Orós et al. [18]. However, in this study, necrotizing enteritis was related to intestinal compaction due to ingestion of anthropogenic waste.

Symptoms suggestive of sepsis represented the most frequent cause of death observed in these animals. The coelomitis symptoms were associated with ulcerative gastritis, perforated ulcerative enteritis, and necrotizing enteritis. It is feasible to assume that the lesion caused in the GI tract wall allowed the translocation of bacteria to the bloodstream and coelomic cavity [18,58,69,70].

Jerdy et al. [70] reported alterations in the digestive system caused by marine debris in 777 *C. mydas* and reported a case of gastric impaction, a finding that was also observed in our study in an animal that presented obstruction from the esophageal segment to the small intestine. According to the aforementioned authors, this type of occurrence is uncommon because the greatest involvement is in the intestinal segments [68].

Inadequate waste disposal due to the lack of effective management in the country is a factor that contributes to the large-scale release of this waste into the marine environment [71–75]. This information corroborates the findings of our study, in which most household plastics identified were categorized as thin plastic, which includes plastic bags, and the “fragments, others, and paper” types, including PET bottles and caps, rigid plastics, elastic bands, rubber bands, and laminated candy packaging. Andrades and colleagues [76] conducted a comprehensive study on anthropogenic waste found on 44 beaches along the Brazilian coast and observed that plastics, cigarette butts and paper accounted for more than 85% of the items collected on the beaches. These data reinforce the importance of studies on the ingestion of waste by animals in this region, especially since it is a nesting and feeding area for turtles [77].

Skeletal system disorders are commonly associated with trauma resulting from interaction with fishing, with fin injuries, erosions, and fractures in the carapace and/or plastron being the most frequent [18]. In this study, the highest frequency of fractures was in the forelimb (27.7%) in 18/61 affected animals. Microscopic lesions of little relevance can also be observed in animals that have interacted with fishing [17].

Regarding the respiratory system, the bilateral lung lesions observed in the present study differ from those in the literature. Some studies report a greater number of cases of unilateral pneumonia, resulting from probable aspiration or piercing trauma to the carapace [18]. Severe lung injury was observed in one of the animals that presented asymmetric lungs, where marked emphysema and pulmonary edema were observed in the right lung and the left lung was reduced, atelectatic and intensely hemorrhagic; in this region the coelomic cavity was edematous and when the peritoneum was removed, there was an extensive hematoma and hemorrhage in the periosteum. These changes are probably the result of interaction with fishing, as reported in the clinical history, or the animal was attacked by a predator. Anthropogenic impacts played a major role in compromising sea turtle health and driving mortality [3,18].

It is noteworthy that seven of the necropsied animals had information in the clinical report of interaction with fishing in various forms, such as carapace fracture due to a probable collision with a boat, cases of necrotizing myositis were associated with animals undergoing clinical treatment, and others that had marks similar to nets on their fins. In these animals, solid residues were observed in the GI tract at necropsy, mostly characterized by nylon monofilaments and fishing lines.

A total of 18/61 animals had some type of interaction with fishing; these injuries ranged from ulcerative dermatitis due to net marks, fractures in fins, rhamphotheca, and shell, as well as hook insertion marks in the oral cavity. These data are in line with those described by Orós and colleagues [18]. It is important to emphasize that the number concerning the cause of death of sea turtles may be underestimated since in most cases there are no specific injuries in the integumentary and respiratory systems, due to the characteristics of the skin structure, which is highly keratinized, not always revealing the injuries, and also the difficulty in identifying decompression injuries [78,79]. Another important factor is the advanced state of autolysis of the cadaver due to the time from death to stranding and its location [13,79].

A study conducted by Tagliolato et al. [80] in southeastern Brazil on the spatial-temporal distribution of sea turtle strandings and factors contributing to mortality showed that the main natural causes of strandings were chronic diseases, endoparasites, and fibropapillomatosis. However, in this same study, the authors report that anthropogenic threats such as the interaction of these animals with fishing and marine debris are present in the region and contribute as factors to their mortality. Other studies also report anthropogenic action as one of the threats to turtles and cite ocean pollution, habitat loss, and climate change as some of the main conservation problems [81–83].

The high incidence of fibropapillomatosis, parasitism, and anthropogenic action corroborates the studies of Glazebrook and Campbell [31]; Gordon et al. [33]; Orós et al. [18,69]; Foley et al. [84]; Jacobson et al. [85] regarding the causes of turtle strandings. However, other authors did not report the predominance or presence of these causes [86]. Future research may provide data on potential correlations between the identified diseases and anthropogenic interactions observed in the present study, aiming to investigate the predisposition of sick animals to incidental capture, among other interactions.

## Conclusion

The results presented here reflect the impact of infectious diseases and anthropogenic action on sea turtles, with a high incidence of animals affected by parasitic diseases, fibropapillomatosis and anthropogenic activities (such as fishing and interaction with solid waste), which are the main factors involved in the cause of death of these animals on the northern coast of Bahia. The importance of the negative effects that anthropogenic action has, directly and indirectly, on the maintenance of life in the marine environment is reiterated. To the authors’ knowledge, this is the first study on the northern coast of Bahia that addresses common pathological processes that affect sea turtles in this region, which is an important feeding and nesting area for these animals.
